# Effects of COVID-19 on the Liver and Mortality in Patients with SARS-CoV-2 Pneumonia Caused by Delta and Non-Delta Variants: An Analysis in a Single Centre

**DOI:** 10.3390/ph17010003

**Published:** 2023-12-19

**Authors:** Monica Muntean, Violeta Briciu, Mihaela Lupse, Doina Colcear, Raul Vlad Macicasan, Agnes Csiszer, Alexandra Manole, Amanda Radulescu

**Affiliations:** 1 Department of Infectious Diseases and Epidemiology, The “Iuliu Hatieganu” University of Medicine and Pharmacy, 400348 Cluj-Napoca, Romania; monica.muntean@umfcluj.ro (M.M.); mihaela.lupse@yahoo.com (M.L.); raulvlad.macicasan@gmail.com (R.V.M.); csiszerdagnes@gmail.com (A.C.); manole_alexandra20@yahoo.com (A.M.); aradulescu@umfcluj.ro (A.R.); 2 The Teaching Hospital of Infectious Diseases, 400348 Cluj-Napoca, Romania; colceardoina@gmail.com

**Keywords:** COVID-19, liver dysfunction, SARS-CoV-2 variants, comorbidities, outcome

## Abstract

The aim of this study was to ascertain patient characteristics, outcomes, and liver injuries in patients infected with different SARS-CoV-2 variants. Data from consecutive adult patients with severe/critical COVID-19 admitted to our hospital during the peak month of the Delta wave were compared to the ancestral, Alpha, and Omicron waves. The dataset of 551 hospitalized patients was similar in the Delta/non-Delta waves. At admission and discharge, the median aminotransferase levels were normal or slightly increased. During the Delta wave (172 vs. 379 non-Delta patients), more patients died (OR 1.69, 95%CI 1.09–2.56) or had liver injury at discharge (alanine aminotransferase, ALT ≥ 2 ULN) (OR 1.97, 95%CI 1.08–3.54). In-hospital mortality was associated with age, lung injury, intensive care unit admission, number of and cardiovascular comorbidities, diabetes, chronic kidney disease, and all inflammatory biomarkers. Serious liver injury at admission (ALT ≥ 5 × ULN) was significantly associated with in-hospital mortality (OR = 7.9, 95%CI 2–28.9). At discharge, drug-induced liver injury (DILI) was found in patients treated with remdesivir, ALT ≥ 2 ULN (OR = 2.62, 95%CI 1.22–5.75). Treatment with dexamethasone, remdesivir, and immunomodulators showed improved survival, OR = 0.50 (95%CI 0.33–0.77). Regardless of the variant and treatment options, less than 2% of patients displayed serious liver injury, which was not found to be a death predictor in multivariable analysis.

## 1. Introduction

The liver is the organ most commonly affected by coronavirus-19 (COVID-19), after the lung [1]. The angiotensin-converting enzyme 2 (ACE2), receptor of the SARS-CoV-2 virus, seems to be mostly expressed in cholangiocytes (59.7%) and less in hepatocytes (2.6%) [2,3]. The most frequent manifestation is a mild increase in alanine aminotransferase (ALT) and aspartate aminotransferase (AST), although the reported incidence from many studies varies between 3.75% and 76.3% [4,5]. These investigations focused on the ancestral strain or Alpha or Beta variants, while the impact on the liver in patients infected with the Delta and Omicron variants is unknown [6,7]. The elevation of alkaline phosphatase (AF), gamma-glutamyl transferase (GGT), and hepatic steatosis are also described in COVID-19 patients [8]. Increased liver enzymes are more frequently described in males and in severe forms. Low albumin is a marker of severity and poor outcomes [9]. The incidence of acute or acute-on-chronic liver failure (ACLF) in COVID-19 patients with chronic liver disease (CLD) was widely reported (10% to 50%), with a high mortality rate of up to 65% [10,11,12]. Patients with a SARS-CoV-2 infection may develop hepatic manifestations due to direct viral action, drug-induced liver injury (DILI), hyperinflammation, the hypoxic-ischemic mechanism [12,13], or exacerbation of associated liver disease; yet, it has not been possible to separate the impact of each mechanism [5]. The complex interplay between hyperinflammation and endothelial cell dysfunction has been evaluated, after administration of convalescent plasma and other potential beneficial molecules, but showed conflicting results [14,15].

Patients who died from COVID-19 presented primarily with steatosis and mild lobular or portal inflammation in liver histology [16]. There is still uncertainty on how pre-existing chronic liver illness may affect SARS-CoV-2 hepatotropism, and no research has so far investigated the histological changes in COVID-19 patients with pre-existing chronic liver disease [17]. Autopsy examinations showed that DILI may be present in patients with COVID-19 and be associated with the widespread use of hepatotoxic medications such as acetaminophen, antivirals (remdesivir, lopinavir-ritonavir), corticosteroids, immunomodulators (tocilizumab), and some antibiotics [1].

The aim of this study was to analyze the differences in disease outcomes and liver injury in consecutive patients with a severe or critical SARS-CoV-2 infection hospitalized during the peak month of the Delta wave and a combined comparator represented by the other peak months during the ancestral, Alpha, and Omicron waves. We were aware that the Delta wave was the most severe, and we aimed to determine whether liver injury was likewise more severe and associated with poor outcomes in the Delta wave compared with the other waves.

Based on published research and our observation that the liver test abnormalities reverted to around baseline levels at discharge, our assumption was that regardless of variant and treatment options, liver derangements were more an indirect expression of severe systemic inflammation rather than a negative key prognostic factor.

## 2. Results

Of 1814 COVID-19 patients admitted during the four study periods, 551 consecutive cases fulfilled the criteria for severe and critical COVID-19; 172 patients were admitted during the peak month of the Delta variant (B.1.617.2) wave (October 2021) and were compared with 379 patients hospitalized in the other three variant peak months (Table 1).

All patients received dexamethasone (5–10 days), prophylactic low-molecular-weight heparin (LMWH), and antiviral treatment. Regarding the antiviral treatment, five-day remdesivir +/− favipiravir (following remdesivir) was considered in 287/551 (52%) patients, only favipiravir (7–14 days) in 27% (149/551) of patients, and hydroxychloroquine in 21% (116/551) of patients. The immunomodulators anakinra (7–10 days) and/or tocilizumab (1–2 doses) were considered in 47% of cases (258/551).

### 2.1. Patient Comparison in the Delta and Non-Delta Waves

Patients’ characteristics, laboratory data, and treatment options are presented in Table 1 and Table 2 (Delta peak month vs. the peak months of the non-Delta waves). 

The Delta wave showed more severity, reflected in significantly higher mortality and length of stay. Overall, 27.4% (151/551) of patients were admitted to the ICU. Age, sex, comorbidities (except cancers), laboratory data (absolute lymphocyte count, CRP, ferritin, LDH, d-dimer, aminotransferases), and antiviral and immunomodulatory treatment use did not differ significantly in the Delta wave compared with the other waves. The most common comorbidities were hypertension (64.4%) and obesity/overweight (52%). Documented chronic liver disease was present in 4.7% (26/551) of patients, and only three patients were newly or previously diagnosed with HBV or HCV infection. Among all patients, at admission, AST, ALT, and GGT were above the ULN in 32% (178/551), 35% (192/551), and 72.8% (164/225), respectively. Comparing both AST and ALT levels above the ULN and <2 ULN, between the Delta and non-Delta waves at admission, we found a significant difference, *p* < 0.0001 and *p* = 0.04, respectively.

At admission, 9% of patients (50/551) presented with moderate/severe liver injury confirmed by aminotransferases (AST) of ≥2 ULN with no significant difference between the variants. However, the percentage of patients with AST levels of ≥2 ULN increased at 7 days and discharge, at 19% and 20%, respectively, without significant differences between the variants. Increased ALT levels were observed mainly at discharge in the Delta wave in patients treated with remdesivir (OR = 2.57 95% CI [1.18 to 5.28]). 

At admission, five patients presented with acute liver injury (ALI) with AST or ALT of ≥10 times ULN, two were previously diagnosed with non-viral liver cirrhosis, and four died. 

At admission, almost half of the tested patients presented with GGT ≥ 2 ULN (46.2%, 104/225—measured mainly in those with increased AST/ALT) with a significant difference between the Delta and non-Delta waves (OR = 1.89, 95% CI [1.055 to 3.34]) which was not found to be significant at discharge (Table 2). A mildly elevated total bilirubin level was found at admission (the highest value detected was 1.51 mg/dL) in 6/211 patients (2.8%).

### 2.2. Patient Comparison between Non-Survivors and Survivors

Among the study patients, the in-hospital mortality of severe/critical cases was 21% (116/551). 

In the univariate analysis, the risk of mortality was associated with age ≥ 65 years, >50% lung damage, intensive care unit (ICU) admission, >2 comorbidities, and >2 cardiovascular or other comorbidities (hypertension, type II diabetes mellitus, chronic kidney disease). Treatments with remdesivir (OR = 0.62 [0.4 to 0.95] *p* = 0.028) or with antivirals and immunomodulators were associated with decreased mortality (OR = 0.5 [0.33 to 0.77] *p* = 0.002) (Table 3).

Laboratory data (survivors and non-survivors) at admission and discharge are presented in Table 4 and Table 5. 

**Figure 1:**
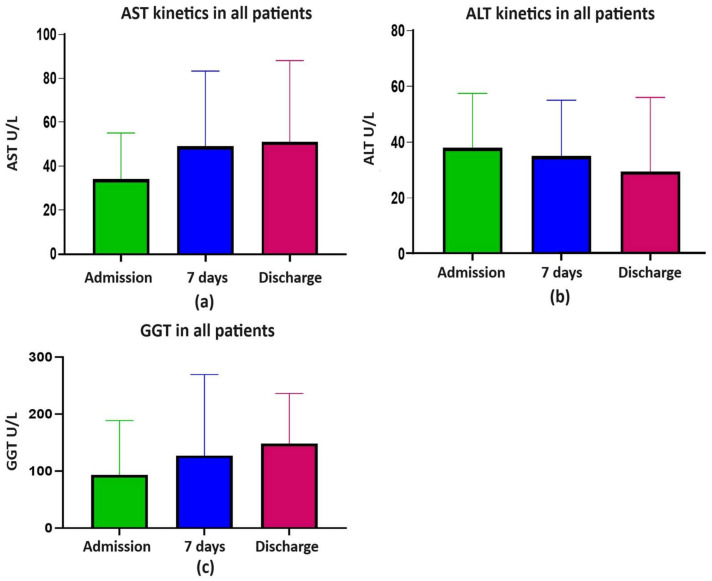
(**a**) AST kinetics; (**b**) ALT kinetics; (**c**) GGT kinetics; ALT, alanine aminotransferase; AST, aspartate aminotransferase; GGT, gamma-glutamyl transferase.

In all patients, the AST levels at admission significantly increased at 7 days and discharge (Welch’s ANOVA, *p* < 0.0001), but for the ALT and GGT levels no significant increases were observed (Welch’s ANOVA, NS) (Figure 1a–c). 

The Spearman’s correlations between the liver enzymes (AST, ALT, and GGT) were almost always highly significant (*p* < 0.01) between them and within hospitalization thresholds (admission, 7 days, and discharge) (Supplementary Table S1). 

**Figure 2:**
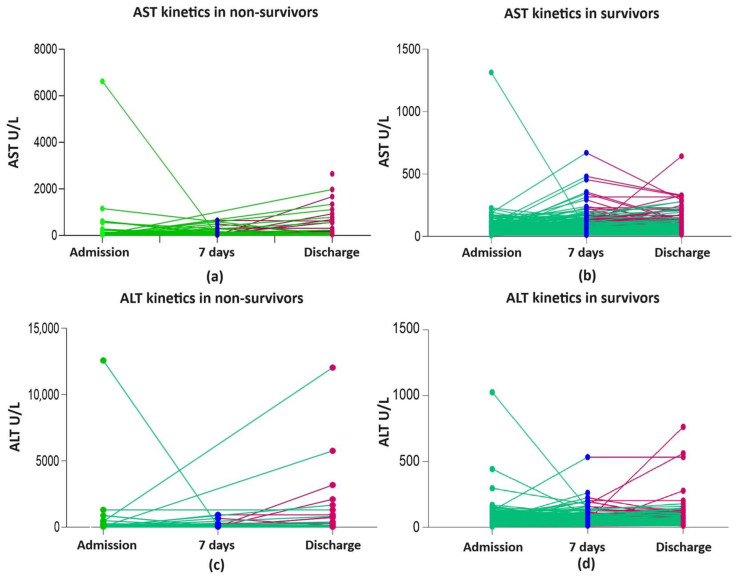
Liver enzyme kinetics in non-survivors and survivors: (**a**) AST kinetics in non-survivors; (**b**) AST kinetics in survivors; (**c**) ALT kinetics in non-survivors; (**d**) ALT kinetics in survivors; ALT, alanine aminotransferase; AST, aspartate aminotransferase; green line shows the kinetics of liver enzymes between admission and day 7 of hospitalization/discharge; red line shows the kinetics of liver enzymes between day 7 and discharge.

At admission, AST and ALT (M and IQR) were within the normal range. Overall, 23.6%, 28%, and 27.1% of all patients presented with AST, ALT, and GGT levels of >50 and <100 ULN, respectively. The risk of death was significantly associated with serious liver damage ranked by a multiplication factor of ≥ 5 for AST and ALT but not for cholestasis enzymes. Overall, significant liver damage, evaluated by AST ≥ 5 ULN, was found in 1.45% (8/551) of patients at admission and in 5.2% (22/420) at discharge (*p* = 0.0008) and for ALT ≥ 5 ULN in 1.6% (9/551) at admission and in 4% (17/420) at discharge (*p* < 0.021) (Table 4, Supplementary Table S1, and Figure 2a–d). As can be observed in the figures, there were few highly elevated transaminases in survivors and non-survivors.

In 25 survivors with elevated ALT (2–5 ULN) at admission (5.4%, 25/435), 13 were treated with remdesivir (according to the protocol, AST and ALT < 5 ULN), and only two had ALT ≥ 2 ULN (maximum 157 U/L) at discharge. None of the four non-survivors with elevated ALT (2–5 ULN) at admission and treated with remdesivir developed a more severe liver injury. Still, in univariate analysis, ALT ≥ 2 ULN and remdesivir treatment were significantly associated with death OR 2.62 (1.22 to 5.75) (Table 5). Elevated GGT was observed, but there was no significant difference between survivors and non-survivors. All inflammatory and prothrombotic laboratory variables were significantly elevated in non-survivors.

At discharge, a similar pattern was observed for all inflammatory and coagulation variables (Table 5). 

In the multivariate analysis, risk of mortality was associated with age, aOR 2.31 (95% CI 1.81–3.00, *p* < 0.001), the total number of comorbidities, aOR 1.29 (95% CI [1.14 to 1.47], *p* < 0.001); LDH > 400 U/L, aOR 2.58 (95% CI [1.62 to 4.23], *p* < 0.001), and Il-6 > 100 pg/mL, aOR 1.37 (95% CI [1.18 to 1.59], *p* < 0.001).

## 3. Discussion

### 3.1. Patient Comparison in the Delta and Non-Delta Waves

Several studies demonstrated direct liver infection by SARS-CoV-2, with primarily expression for ACE2 in cholangiocytes and hepatocytes [17,18,19,20,21,22]. Mainly initial studies showed a noteworthy association between liver injury and poor prognosis [23,24,25], while others found no association [10,26,27], but the conclusions remain elusive even in meta-analyses [28,29,30]. 

Our study found that mild liver injury (1–2 times above the upper limit of normal (ULN)) is relatively common in severe and critical SARS-CoV-2 infected patients, as presented in Table 2. 

The comparison between the Delta wave and the other waves did not show many differences except for a higher rate of death, LOS, and an overall mortality rate of 21%. We did not find any difference between the waves regarding age, gender, pre-existing conditions (except cancers more frequently encountered in the non-Delta waves), and laboratory parameters at admission. Similar to our results, Laffont-Lozes et al., assessing the factors associated with mortality in severe COVID-19 patients hospitalized in Nimes University Hospital, France, during the Delta and Omicron waves, found an in-hospital death rate of 22% [22]. The high in-hospital mortality rate is explained by the selection of severe/critical cases, while a previous study published by our center comparing a total of 2235 COVID-19 patients hospitalized during the Delta and Omicron waves found a mortality rate of 6.58% and 3.48% in the Delta and Omicron waves, respectively [31]. 

Since half of the patients were obese/overweight, one third had type II diabetes, and presumably non-alcoholic fatty liver disease (NAFLD) and hepatocellular damage may already have existed before admission. 

In a recently published study regarding SARS-CoV-2 and the liver, Luxenberger et al. suggest that the probable mechanisms contributing to more severe disease in patients with NAFLD involve the impairments of T cell immunity and adipokine imbalance, as well as leptin and adiponectin, which might suppress T cell-mediated immune responses [20]. NAFLD does not seem to be a driver for mortality in these patients, as Marjot et al. found a negative association for mortality in patients with NAFLD (OR 0.55; 95% CI [0.38 to 0.81]; *p* = 0.002), whereas alcohol-related liver disease showed a positive association with death (OR 3.11; 95% CI [2.12 to 4.55]; *p* < 0.001) [17]. Conversely, Roca-Fernandez et al. showed that obese individuals with COVID-19 and fatty liver disease had a higher risk of hospitalization [32]. Broadly stated, Ebrahimi et al. discovered an elevated risk of serious infections, primarily respiratory (aHR, 1.54; 95% CI [1.40 to 1.70]), in a large Swedish prospective cohort of individuals with biopsy-proven NAFLD [33]. 

Patients with liver cirrhosis are more susceptible to infections, including COVID-19, when compared with the general population, which is explained by immune dysfunction, high levels of proinflammatory cytokines, coagulopathy, and DILI [18,20,34]. Mortality among patients with chronic liver disease (CLD) or cirrhosis following SARS-CoV-2 infection was found to increase with each Child–Pugh (CP) class from 8% in CLD to 51% in CP-C [17,35]. In a case series from Romania, in patients with liver cirrhosis with or without COVID-19 (45 and 100, respectively), in-hospital mortality was found to be as high as 46.7% versus 15%, and was associated with infections, pulmonary injury, and acute-on-chronic liver failure (ACLF) [36]. 

In our study, all but 2 of the 26 patients who had non-viral liver cirrhosis or chronic liver disease displayed moderately to severely increased transaminase values (<250 U/L) on admission. These two individuals experienced ACLF; one of them died after being admitted with AST and ALT levels greater than 1000 U/L, most likely due to the SARS-CoV-2 infection. Similar to our results, in a Romanian teaching hospital, Moga et al. found mildly elevated median transaminase levels at admission in cirrhotic patients with or without COVID-19 [36].

Regarding the possible effect of medication on liver function, DILI might be considered, mainly in the Delta wave, since we observed an increase in ALT ≥ 2 ULN from 8.7% (15/172) to 17.5% (25/143) at discharge, and after remdesivir treatment. Nonetheless, other causes of elevated liver enzymes rather than liver injury, such as cardiac injury, ischemia, and hyperinflammation, might be involved [37]. 

In line with previous research, and since there were no differences in age, sex, underlying disease, or liver enzymes (except elevated ALT at discharge) between the Delta and non-Delta waves, our results suggest that hepatocellular injury was not a significant driver for increased mortality in the Delta wave.

### 3.2. Prognosis and Liver Injury

COVID-19 death predictors have been extensively evaluated in large databases all over the world, with age being the most important factor along with male sex, number of preexisting diseases, and many comorbidities like cardiovascular and/or cerebrovascular, obesity, diabetes, and dementia [11,38,39,40,41,42]. Fortunately, even in immunocompromised patients, SARS-CoV-2 infection severity was much lower in the Omicron wave compared with the previous waves, as demonstrated by the WHO ISARIC UK prospective cohort study [43].

Liver disease was not found significant in a population-based cohort analysis within the OpenSAFELY platform in the United Kingdom [38], while in a large US National COVID cohort, clinical severity was associated with liver disease (OR = 1.20, 95%CI [1.08 to 1.34]) [39].

Regarding the risk of in-hospital mortality, our data are consistent with a previous report published by Radulescu et al. on COVID-19 fatalities, showing that age, comorbidity index > 2, cardiovascular comorbidities, hypertension, chronic kidney disease, and type II diabetes mellitus were associated with a higher risk of poor outcomes [44].

Somehow surprisingly, our results did not find an association between obesity and in-hospital mortality, which may be explained by the high rate of obesity (52%). By the end of our study (February 2022), obesity and diabetes mellitus were present in 18.6% and 29.6%, respectively, of all COVID-19-related deaths in Romania [44]. 

Despite many studies that have confirmed male gender as being associated with more severe COVID-19 and a higher rate of death [23,45,46,47], our study found no association between male gender and outcomes; older age (>65 years) was associated with death similar to other reports [41,48]. Our findings are consistent with data reporting that underlying diseases are more likely to affect COVID-19 severity than liver damage [39,42,49]. During three pandemic years, between 27 February 2020 and 31 March 2023, there were 9049 patients hospitalized in our medical unit, with a low vaccination rate of 16.42% [50], therefore, we did not assess the vaccine protective effect in our study group.

Since the beginning of the pandemic, a great body of knowledge has shown that poor outcomes in hospitalized COVID-19 patients are associated with biomarkers like decreased lymphocyte and platelet count, elevated C reactive protein, procalcitonin, D-dimer, lactate dehydrogenase, aminotransferases, creatine kinase, and creatinine levels [51,52,53]. Proinflammatory and prothrombotic states in severe COVID-19 have been evaluated and increased ferritin and Il-6 were shown to be associated with poor outcomes [39,54,55]. Similarly, we found that the proinflammatory and prothrombotic biomarkers were abnormal in all patients, at admission and discharge, as median and IQR values, which is not unexpected since all the patients were hospitalized for severe and critical disease. Moreover, at admission, all the biomarkers above the established cut-offs (including AST and ALT ≥ 5 ULN) were significantly associated with in-hospital mortality. In multivariate analysis, increased LDH and Il-6 were significantly associated with poor prognoses. 

At discharge, the kinetics of all the biomarkers were associated with poor outcomes. Despite the fact that highly elevated ferritin was found in non-survivors, it did not turn out to be significant in multivariable analysis; still, it has been shown that its level could be correlated with the IL-6 levels [56]. Ferritin also depends on IL-18 levels, explaining why ferritin levels did not significantly decrease with or without IL-6 blockade, neither in survivors nor in non-survivors, as presented in our previous publication [57]. 

Elevated liver enzymes (ALT and AST) have been detected in the range of 14–76% of COVID-19 patients in different studies [6,10,35]. AST is an enzyme less specific for liver injury than ALT due to additional extrahepatic synthesis. Elevated AST levels appear earlier, with more rapid and pronounced increases than ALT levels. At admission and discharge, the median values of transaminases were within the normal range, similar to other published studies [30,37,58,59]. The frequency of moderate/severe ALI (AST and ALT levels of ≥2 or ≥5 ULN) was similar regardless of the variant (~9%, ~2%, respectively) and at 7 days, the increase was mainly for AST ≥ 2 ULN (19%) and less for ALT ≥ 2 ULN (8.7%). 

At admission, we found similar percentages of abnormally elevated liver enzymes (AST, ALT, and GGT > ULN), similar to a study performed in a university hospital in Bucharest [4]. They found serious liver damage at admission, as evidenced by AST and ALT levels (5–10 ULN) in 0.77% and 0.25% of patients, respectively, which are similar to our findings of AST and ALT (>5 ULN) in 1.45% and 1.6% patients, respectively. Among the study patients, we have described a significant increase in liver enzymes during hospitalization, suggesting a combined effect between SARS-CoV-2 infection and drug-induced liver injury (DILI), consistent with other authors [10,16,17,20,21,35,60]. 

Chew et al. found that 105 (12.6%) of the 834 consecutive COVID-19 patients admitted to Yale New Haven Hospital had significant liver injury (>5 ULN). However, in multivariate logistic regression, liver injury was not associated with death [16]. 

In a retrospective cohort study in Romania, Crisan et al. found that among 370 consecutive patients with moderate and severe COVID-19 and no preexisting liver disease, 72.9% had liver abnormalities at admission (AST and ALT mildly increased, and median peak values of <5 ULN). However, the risk of death was 11.6%, significantly associated with non-invasive fibrosis estimators (Fibrosis-4) and elevated AST [61]. 

In patients with severe COVID-19, increased GGT and decreased albuminemia have also been documented [37]. In our study, cholestasis (increased GGT and ALP) was found mainly for GGT > ULN at admission in 164/225 (72.8%) and at discharge in 68/75 (90.6%). Relatively similar results were found in the study performed in Bucharest; elevated cholestasis enzymes were found in 45.5% of the patients at admission, and in the majority of their patients (87.5%), an isolated increase in GGT was reported. We rarely found mildly elevated bilirubin (2.8%), comparable with the value found at admission in the study from Bucharest, of 1.6% of patients [4].

Nonetheless, many experts agree with the fact that in most cases, liver injury is mild and transient during COVID-19 but may be associated with poor prognoses [23,25,35,46,62].

### 3.3. DILI

Besides antivirals, updated guidelines strongly recommend in severe or critical COVID-19: systemic corticosteroids; IL-6 receptor blockers (tocilizumab, sarilumab); and the Janus kinase (JAK) inhibitor baricitinib (in addition to corticosteroids) [63]. 

Several drugs can cause DILI, defined by an increase in liver enzymes with or without symptoms, the most common being the hepatocellular pattern. DILI, usually mild, may occur through intrinsic (dose-dependent) or idiosyncratic mechanisms [17,60]. 

According to international and national protocols, well-known drugs with the highest risk of DILI, such as acetaminophen, antibiotics (amoxicillin-clavulanate, azitromycin), and NSAIDs, were not considered for our patients. Dexamethasone, LMWH, hydroxycloroquine, and anakinra are associated with a low risk of DILI; instead, tocilizumab, remdesivir, and favipiravir may cause mainly hepatocellular injuries [60,64,65]. 

In a meta-analysis of 20,874 patients, Kulkarni et al. estimated a 25.4% incidence of DILI in COVID-19 patients based on increases in liver enzymes and bilirubin but without convincing evidence besides the temporal relationship [30]. 

Tuteja et al. evaluated in a large retrospective pharmacogenetic study the probable mechanisms involved in DILI associated with remdesivir. Compared with the FDA report (11.7% incidence of liver enzyme increase among patients enrolled in the compassionate use program) [66], the authors found a 30% increase in ALT during hospitalization associated with remdesivir use [67]. The elevation was higher in non-Hispanic white individuals with CYP2C19 intermediate/poor metabolizer phenotypes compared with individuals with normal or rapid metabolizer phenotypes, but a definite conclusion was not drawn [67]. We cannot preclude DILI being associated with remdesivir, but the low median values of ALT in evolution do not represent a strong basis for causal association. However, at discharge, elevated ALT levels were more likely associated with poor prognoses compared with AST, suggesting DILI interplay. Nevertheless, remdesivir treatment showed improved survival, as observed in many studies supporting the guidelines [67,68].

Chew et al. found that tocilizumab use was an independent predictor of liver injury but the study conclusion was that “liver test abnormalities known to be associated with COVID-19 are secondary to other insults, mostly ischemia or drug-induced liver injury, and do not lead to liver insufficiency or death” [16]. 

Laffont-Lozes et al. suggest a protective effect on mortality in Delta and Omicron waves if tocilizumab was given in the first 48 h after hospital admission, but with no significant differences in mortality between waves, similar to our results [22]. 

As in other studies, we did not observe severe liver injury except for a moderate increase in ALT values at discharge [39,69,70]. We did not find a protective effect on mortality if tocilizumab was considered, but treatment with dexamethasone, antivirals, and immunomodulators (tocilizumab or anakinra) was associated with improved survival (OR = 0.50 (0.33–0.77), *p* = 0.002).

Favipiravir is an antiviral against influenza viruses and filoviruses used in COVID-19-infected patients in many countries, including Romania. In a recent meta-analysis including eight randomized controlled trials, Batool et al. found no significant differences in terms of viral clearance, clinical improvement, ICU need, or need for oxygen therapy, but there were no safety issues [71]. 

Shah et al. conducted a multicenter trial on favipiravir in patients who were admitted to hospital with COVID-19, with 499 patients assigned to treatment and standard of care. They did not find differences in terms of time to recovery, but a faster recovery was found in patients under the age of 60 years, and no difference between groups for serious adverse events [72]. Likewise, in the univariate analysis, we did not find a difference between survivors and non-survivors in association with favipiravir use, but no conclusions could be reached regarding safety issues.

### 3.4. Study Limitations and Strengths

The strength of our study is represented by the assessment of patients in the peak months of the SARS-CoV-2 waves and the analysis of in-hospital mortality predictors, including laboratory tests in dynamics. In addition, we classified the liver injury as mild, moderate, and severe, highlighting the impact on prognosis.

We appreciate that there were more similarities than differences between the waves, considering clinicians’ competence, intense pressure, and treatment apart from antivirals. Even though the antiviral treatment was not homogenously ensured during the entire study period, oxygen therapy, dexamethasone, and anticoagulants were considered in all severe and critical patients from the beginning of the pandemic, and remdesivir was included as an antiviral treatment immediately after the guidelines were released.

This study has limitations inherent in an observational study of a relatively modest number of cases from a referral COVID-19 hospital setting. The retrospective observational nature of our study precludes making a causation claim and implies possible alternative explanations. The variant was not tested in each patient, yet according to the national surveillance data, during the peak month of each wave, a unique variant was identified. The interference between liver injury due to severe SARS-CoV-2 infection and DILI could not be properly assessed since we did not include non-invasive fibrosis estimators, computed tomography-documented liver steatosis, or liver histology, but we believe that these investigations might have harbored little clinical relevance for the purpose of our study.

We included severe patients; therefore, inferences for patients with milder diseases might be biased, though selected cases are more relevant for severity analysis. 

## 4. Materials and Methods

### 4.1. Study Design and Setting

The retrospective study was performed in the Teaching Hospital of Infectious Diseases Cluj-Napoca, Romania. We analyzed data from consecutive adult patients with severe and critical COVID-19 who were hospitalized during the peak months of the first four waves: November 2020 (Wuhan strain), March 2021 (B.1.1.7, Alpha variant), October 2021 (B.1.617.2, Delta variant), and February 2022 (B.1.1.529, Omicron). According to the Romanian National Institute of Public Health, during the peak month of each wave, the dominant variant was almost exclusively present [44]. 

### 4.2. Participants and Variables

The data collected from the hospital’s electronic system included: demographics, clinical data, comorbidities (according to the International Classification of Diseases), relevant laboratory tests (performed at admission, day 7, discharge/death), chest computed tomography (CT), serological tests for hepatitis B and C viruses (HBV and HCV), and intensive care unit (ICU) admission. The inclusion window’s end point was set at in-hospital death or discharge with improved health.

We included in this study the severe and critical cases of COVID-19, according to updated national and international protocols [68,73,74,75,76]: (a) Severe: the presence of any of the following: respiratory rate >30 breaths/min, oxygen saturation SpO_2_ (resting state) ≤ 94%, partial arterial oxygen pressure (PaO_2_)/fraction of inspired oxygen (FiO_2_) ≤ 300 mmHg; (b) Critical: the presence of any of the following: respiratory failure that requires invasive mechanical ventilation, shock, or any organ failure that needs ICU admission. The definition of abnormal liver tests was based on reference laboratory standards. 

ALI was ranked as mild if aminotransferases were above the upper limit of normal (ULN) but <2 ULN, and serious if ≥5 ULN. We defined moderate/severe liver injury as ≥2 ULN for aminotransferases (alanine, aspartate aminotransferase; ALT, AST respectively; ULN 45 U/L), gamma-glutamyl transferase (GGT; ULN 50 U/L), and alkaline phosphatase (ALP; ULN 105 U/L). Thus, moderate/severe liver injury was defined by ALT or AST ≥ 100 U/L, GGT ≥ 100 U/L, and ALP ≥210 U/L. The threshold of ALT or AST ≥ 5 ULN is in accordance with the test that can assist in the diagnosis of DILI, the Roussel Uclaf Causality Assessment Method (RUCAM), defined by a serum ALT above five times the ULN and/or a serum ALP (alkaline phosphatase) above two times the ULN [77,78]. 

Also, we included the following predictors for severe disease: IL-6 > 100 pg/mL (ULN 6.4 pg/mL), C-reactive protein (CRP) > 75 mg/L (ULN 10 mg/L), ferritin > 500 ng/mL (ULN 336 ng/mL), D-dimer > 1 mg/L (ULN 0.55 mg/L), and lactate dehydrogenase (LDH) > 400 U/L (ULN 250 U/L) [73,74,75,76]. SARS-CoV-2 infection was confirmed by reverse transcriptase-polymerase chain reaction (RT-PCR) tests or rapid antigen tests performed on nasopharyngeal swabs. 

For grading lung involvement, the French Society of Thoracic Imaging recommendations were used, based on visual assessment: minimal (<10%), moderate (10–25%), extensive (25–50%), severe (50–75%), and critical (>75%), combined with the Örebro COVID-19 Scale (ÖCoS) for the parenchymal pattern [79]. The worst lung damage during hospitalization was recorded.

The national treatment protocols for severe and critical cases included: oxygen by nasal cannula, high flow or invasive/noninvasive mechanical ventilation, antivirals (remdesivir, favipiravir, hydroxychloroquine, molnupiravir), anticoagulants, immunomodulators (dexamethasone, tocilizumab, and anakinra) and antibiotics for bacterial superinfection [73]. 

This study was approved by the hospital’s ethics committee (approval number: 10985/10.06.2022).

### 4.3. Statistical Analysis

Descriptive statistics were used on the study population to describe their general and baseline characteristics, demographics, and past history. The Kolmogorov–Smirnov test was used to assess the distribution of quantitative variables. Data with a non-normal distribution were presented using the median, and selected centiles (25th–75th). Categorical variables were presented as numbers and proportions. Hypothesis testing was performed using the Fisher exact test (for comparing qualitative variables) or Mann–Whitney tests (for comparing two independent groups concerning non-normally distributed quantitative variables). The Spearman correlation coefficient was employed to assess the relationship between two quantitative non-normally distributed variables. Univariate logistic regression models were fitted to assess the relationship between predictors and the outcomes of interest (Delta vs. non-Delta waves, and mortality). A multivariate logistic regression model was performed with several independent variables to predict their association with mortality. Odds ratios, 95% confidence intervals, and *p*-values were reported for each logistic regression model. For all statistical tests, a 0.05 level of statistical significance was used. Statistical analysis was performed with GraphPad Prism version 10.0.0, and with R version 4.0; *p*-values of <0.05 were considered to be statistically significant [80].

## 5. Conclusions

Liver injury is a common extrapulmonary manifestation in hospitalized patients with SARS-CoV-2 infections. Despite the abundance of published data evaluating liver tests in COVID-19 patients, it is still difficult to draw conclusions on the effect of SARS-CoV-2 virus infection on the liver and on the prevalence of elevated liver enzymes.

Though our study has the limitations inherent in a retrospective observational study of a relatively modest number of cases, in severe/critical COVID-19 patients, we reported a high in-hospital mortality rate associated with age, comorbidities, and lung damage, regardless of variant. Patients with abnormal liver biochemistry at admission might be susceptible to an unfavorable outcome, but serious liver dysfunction was rarely observed during hospitalization and we did not find a worse prognosis related to the Delta variant as we presumed. We found an association between increased liver enzymes and remdesivir but the interference between liver injury due to severe SARS-CoV-2 infection and DILI could not be properly assessed. Nevertheless, treatment with dexamethasone, antivirals, and immunomodulators was associated with improved survival.

Clinicians should monitor liver enzymes in patients with severe COVID-19 since medications with potentially hepatotoxic side effects are recommended.

## Figures and Tables

**Table 1:** Univariate analysis of demographic and clinical data (Delta peak month versus the peak months within the waves from 2020 to 2022).

Variable	Details	Delta N = 172 (31.2%)	Non-Delta N = 379 (68.8%)	Total N = 551	Statistics
Age	M (IQR)	68 (51–76)	67 (57–76)	67 (56–76)	*p* = 0.07
Male gender		85 (49.4)	195 (51.4%)	280 (50.8%)	NS
Length of stay (days) LOS	M (IQR)	13 (9–19)	11 (7–16)	12 (8–17)	MW: *p* = 0.0001
Non-survivors	N (%)	47 (27.3%)	69 (18.2%)	116 (21%)	OR = 1.69 [1.09, 2.56] *p* = 0.0018
ICU admission	N (%)	35 (20.3%)	116 (30.6%)	151 (27.4%)	OR = 0.56 [0.37, 0.89] *p* = 0.013
Lung damage (%)	M (IQR)	50 (40–80)	50 (40–70)	50 (40–70)	MW: NS
Cardiovascular comorbidities (>2)	N (%)	29 (16.9%)	66 (17.4%)	95 (17.2%)	OR = 0.96 [0.59, 1.55] NS
Hypertension	N (%)	107 (62.2%)	248 (65.4%)	355 (64.4%)	OR = 0.86 [0.59, 1.26] NS
Diabetes type II	N (%)	62 (36%)	122 (32.2%)	184 (33.4%)	OR = 1.18 [0.81, 1.74] NS
Obesity/overweight	N (%)	84 (49%)	202 (53.3%)	286 (52%)	OR = 0.83 [0.58, 1.19] NS
Chronic kidney disease/Hemodialysis	N (%)	14 (8%)	33 (8.7%)	47 (8.5%)	OR = 0.92 [0.49, 1.79] NS
COPD/asthma	N (%)	26 (15%)	42 (11%)	68 (12.3%)	OR = 1.42 [0.83, 2.41] NS
Chronic liver disease/cirrhosis	N (%)	10 (5.8%)	16 (4.2%)	26 (4.7%)	OR = 1.4 [0.64, 3.2] NS
Cancers	N (%)	9 (5.2%)	46 (12.1%)	55 (10%)	OR = 0.39 [0.19, 0.80] *p* = 0.013
Remdesivir use	N (%)	95 (55.2%)	192 (51%)	287 (52%)	OR = 0.83 [0.57, 1.18] NS
Tocilizumab use	N (%)	16 (9.3%)	57 (15%)	73 (13.2%)	OR = 0.57 [0.32, 1.05] NS
Non-survivors and remdesivir treatment	N (%)	28/95 (29.5%)	43/192 (22.4%)	71/287 (24.7%)	OR= 0.69 [0.39, 1.18] NS
Non-survivors and antivirals + immunomodulators	N (%)	17/57 (29.8%)	32/109(29.4%)	49/166 (29.5%)	OR= 0.98 [0.49, 1.92] NS

ULN, upper limit of the normal; CRP, C-reactive protein; ALT, alanine aminotransferase; AST, aspartate aminotransferase; GGT, gamma-glutamyl transferase; LDH, lactate dehydrogenase; CI, confidence interval; IQR, interquartile range; M, median (min:max); MW, Mann–Whitney test; OR, odds ratio; OR (95% CI) and *p*-value from Fisher test; NS, not significant.

**Table 2:** Univariate analysis of laboratory variables in dynamics (Delta wave vs. the other waves).

Variable		Delta N = 172 (31.2%)	Non-Delta N = 379 (68.8%)	Total N = 551	Statistics
CRP > 75 mg/L	N (%)	103/172 (60%)	215/379 (56.7%)	318/551 (58%)	OR = 1.13 [0.79, 1.65] NS
D-dimer > 1 mg/L	N (%)	69/172 (40%)	148 (39%)	217 (39.4%)	OR = 1.06 [0.74, 1.54] NS
Lymphocyte count	M (IQR)	0.66 (0.52–0.99)	0.75 (0.49–1)	0.73 (0.51–1)	MW—NS
Ferritin	M (IQR)	597 (248–1183)	580 (311–1025	585 (290–1025)	MW—NS
LDH	M (IQR)	384 (302–519)	373 (279–459)	376 (283–479)	MW—NS
Abnormal AST < 2 ULN, admission	N (%)	60/172 (34.9%)	70/379 (18.5%)	130/551 (23.6%)	OR = 2.36 [1.56, 3.56] *p* < 0.0001
AST ≥ 2 ULN, admission	N (%)	15/172 (8.7%)	35/379 (9.2%)	50/551 (9%)	OR = 0.93 [0.49, 1.75] NS
AST ≥ 5 ULN, admission	N (%)	2/172 (0.6%)	7/379 (1.8%)	9/551 (1.6%)	OR = 0.6 [0.13, 2.83] NS
Abnormal ALT < 2 ULN, admission	N %)	59/172 (34.3%)	95/379 (25%)	154/551 (28%)	OR = 1.52 [1.03, 2.26] *p* = 0.040
ALT ≥ 2 ULN, admission	N (%)	15/172 (8.7%)	24/379 (6.3%)	39/551 (7%)	OR = 1.41 [0.74, 2.78] NS
ALT ≥ 5 ULN, admission	N (%)	1/172 (0.58%)	8/379 (2.1%)	9/551 (1.6%)	OR = 0.54 [0.11, 2.25] NS
Abnormal GGT < 2 ULN, admission	N (%)	13/70 (18.6%)	48/155 (31%)	61/225 (27.1%)	OR = 0.32 [0.16, 0.44] *p* = 0.0012
GGT ≥ 2 ULN, admission	N (%)	40/70 (57.1%)	64/155 (41.3%)	104/225 (46.2%)	OR = 1.89 [1.05, 3.34] *p* = 0.0308
AST increase ≥ 2 ULN at 7 days	N (%)	20/119 (17%)	41/204 (20%)	61/323 (19%)	OR = 0.77 [0.42, 1.41] NS
ALT increase ≥ 2 ULN at 7 days	N (%)	9/119 (7.5%)	19/204 (9.3%)	28/323 (8.7%)	OR = 0.79 [0.33, 1.80] NS
Abnormal AST < 2 ULN at discharge	N %)	35/143 (24.5%)	86/278 (31%)	121/421 (28.7%)	OR = 0.73 [0.46, 1.41] NS
AST increase ≥ 2 ULN at discharge	N (%)	29/143 (20.2%)	56/278 (20%)	85/421 (20%)	OR = 1.0 [0.61, 1.66] NS
Abnormal ALT < 2 ULN at discharge	N %)	25/143 (17.5%)	52/278 (18.7%)	77/421 (18.3%)	OR = 0.96 [0.57, 1.60] NS
ALT increase ≥ 2 ULN at discharge	N (%)	25/143 (17.5%)	27/278 (10%)	52/421 (12.3%)	OR = 1.97 [1.08, 3.54] *p* = 0.028
GGT increase ≥ 2 ULN at discharge	N (%)	16/23 (69.6%)	37/52 (71.2%)	53/75 (70.7%)	OR= 0.92 [0.34, 2.5] NS
AST ≥ 2 ULN at discharge and remdesivir use	N (%)	17/95 (18%)	24/192 (12.5%)	41/287 (14.3%)	OR = 1.52 [0.75, 2.9] NS
ALT ≥ 2 ULN at discharge and remdesivir use	N (%)	16/95 (17%)	14/192 (7.3%)	30/287 (10.5%)	OR = 2.57 [1.18, 5.28] *p* = 0.022

ULN, upper limit of the normal; CRP, C-reactive protein; ALT, alanine aminotransferase; AST, aspartate aminotransferase; GGT, gamma-glutamyl transferase; LDH, lactate dehydrogenase; CI, confidence interval; IQR, interquartile range; M, median (min:max); MW, Mann–Whitney test; OR, odds ratio; OR (95% CI) and *p*-value from Fisher test; NS, not significant.

**Table 3:** Univariate analysis of selected variables by death in COVID-19 patients.

Variable	Details	Non-Survivors N = 116 (21%)	Survivors N = 435 (79%)	Total N = 551	Statistics
Age	M (IQR)	77 (70–83)	64 (53–73)	67 (56–76)	MW *p* < 0.0001
Age ≥ 65 years	N (%)	102 (88%)	212 (49%)	314 (57%)	OR = 7.66 [4.32, 14.23] *p* < 0.0001
Male gender	N (%)	51 (44%)	229 (52.6%)	280 (51%)	NS
Length of stay (days)	M (IQR)	12 (6–19)	12 (8–17)	12 (8–17)	MW: NS
Lung damage	M (IQR)	70 (50–80)	50 (40–70)	50 (40–70)	MW: NS
Lung damage > 50%	N (%)	90/116 (77.6%)	288/435 (66.2%)	378 (68.6%)	OR = 1.76 [1.10, 2.81] *p* = 0.018
ICU admission	N (%)	68/116 (58.7%)	83/435 (19%)	151 (27.4%)	OR = 6 [3.84, 9.33] *p* = 0.0001
Comorbidities (N)	M (IQR)	3.5 (2–5)	2 (1–3)	2 (1- 4)	MW *p* < 0.0001
Comorbidities > 2	N (%)	83 (71.5%)	170 (39%)	253 (46%)	OR = 3.92 [2.51, 6.13] *p* < 0.0001
Cardiovascular comorbidities (>2)	N (%)	40 (34.5%)	55 (12.6%)	95 (17.2%)	OR = 3.63 [2.24, 5.80] *p* < 0.0001
Diabetes mellitus type II	N (%)	49 (42.2%)	135 (31%)	184 (33.3%)	OR = 1.62 [1.06, 2.44] *p* = 0.026
Obesity/overweight	N (%)	55 (47.4%)	231 (53.1%)	286 (52%)	OR = 0.7 [0.54, 1.2] NS
Hypertension	N (%)	99 (85.3%)	256 (59%)	355 (64.4%)	OR = 4.07 [2.36, 7.13] *p* < 0.0001
Chronic kidney disease/HD	N (%)	23 (20%)	24 (5.5%)	47 (8.5%)	OR = 4.23 [2.3, 7.69] *p* < 0.0001
COPD/asthma	N (%)	20 (17.2%)	48 (11%)	68 (12.3%)	OR = 1.68 [0.96, 2.9] NS
Chronic liver disease/cirrhosis	N (%)	7 (6%)	19 (4.4%)	26 (4.7%)	OR = 1.4 [0.53, 3.45] NS
Cancers	N (%)	15 (13%)	40 (9.2%)	55 (10%)	OR = 1.46 [0.76, 2.7] NS
Remdesivir use	N (%)	71 (61.2%)	216 (49.6%)	287 (52%)	OR = 0.62 [0.4, 0.95] *p* = 0.028
Favipiravir use	N (%)	23/116 (19.8%)	126/435 (29%)	149 (27%)	OR = 0.60 [0.36, 1.001] *p* = 0.0506
Tocilizumab use	N (%)	18 (15.5%)	55 (12.6%)	73 (13.2%)	OR = 0.78 [0.45, 1.42] NS
Antivirals+ immunomodulators	N (%)	49 (42.2%)	117 (26.9%)	166 (30%)	OR =0.50 [0.33, 0.77] *p* = 0.002

ULN, upper limit of the normal; CRP, C-reactive protein; ALT, alanine aminotransferase; AST, aspartate aminotransferase; GGT, gamma-glutamyl transferase; LDH, lactate dehydrogenase; CI, confidence interval; IQR, interquartile range; M, median (min:max); MW, Mann–Whitney test; OR, odds ratio; (95% CI) and *p*-value from Fisher test; NS, not significant.

**Table 4:** Univariate analysis of laboratory variables at admission, non-survivors vs. survivors.

Variable	Details	Non-Survivors N = 116 (21%)	Survivors N = 435 (79%)	Total N = 551	Statistics
D-dimer (mg/L)	M (IQR)	1.2 (0.7–2.3)	0.72 (0.47–1.4)	0.81 (0.49–1.5)	MW: *p* < 0.0001
D-dimer >1 mg/L	N (%)	67/110 (61%)	146/417 (35%)	213/527 (40.4%)	OR = 2.89 [1.88, 4.43] *p* < 0.0001
CRP (mg/L)	M (IQR)	130 (68–230)	83 (47–140)	91 (50–160)	MW: *p* < 0.0001
CRP > 75 mg/L	N (%)	84/116 (72.4%)	234/435 (53.9%)	318/551 (57.7%)	OR = 2.25 [1.44, 3.52] *p* < 0.0003
Lymphocyte N × 10^3^/µL	M (IQR)	0.64 (0.41–0.85)	0.75 (0.54–1.1)	0.73 (0.51–1)	MW: *p* = 0.0001
Lymphocyte <1 × 10^3^/µL	N (%)	97/116 (83.6%)	310/435 (71.3%)	407/551 (74%)	OR =2.06 [1.21, 3.45] *p* = 0.0063
Ferritin (ng/mL)	M (IQR)	756 (299–145)	548 (285–1019)	585 (290–1068)	MW: *p* = 0.017
Il-6 (pg/mL)	M (IQR)	67 (33–135)	25 (11–64)	33 (13–77)	MW: *p* < 0.0001
LDH (U/L)	M (IQR)	441 (313–613)	367 (278–456)	376 (283–479)	MW: *p* < 0.0002
LDH > 400 U/L	M (IQR)	71/116 (61.2%)	155/435 (35.6%)	226/551 (41%)	OR = 2.69 [1.75, 4.06] *p* < 0.0001
AST (U/L)	M (IQR)	35 (24–59)	37 (25–59)	37 (25–59)	MW: NS
AST ≥ 2 ULN	N (%)	16/116 (13.8%)	34/435 (7.8%)	49/551 (8.9%)	OR = 1.90 [0.99, 3.59] NS
AST ≥ 5 ULN	N (%)	7/116 (6%)	1/435 (0.23%)	8/551 (1.45%)	OR = 27.9 [3.83, 314.6] *p* < 0.0001
ALT (U/L)	M (IQR)	43 (24–68)	35 (24–52)	36 (24–54)	MW: NS
ALT ≥ 2 ULN	N (%)	11/116 (9.5%)	28/435 (6.4%)	39/551 (7.1%)	OR = 1.51 [0.72, 3.07] NS
ALT ≥ 5 ULN	N (%)	6/116 (5.5%)	3/435 (0.69%)	9/551 (1.6%)	OR = 7.9 [2, 28.9] *p* = 0.0037
GGT (U/L)	M (IQR)	82 (49–164)	94 (46–196)	93 (46–189)	MW: NS
GGT ≥ 2 ULN	N (%)	26/62 (41.9%)	78/163 (47.9%)	104/225 (46.2%)	OR = 1.32 [0.8, 2.15] NS
GGT ≥ 5 ULN	N (%)	9/62 (14.5%)	33/163 (20.2%)	42/225 (18.7%)	OR = 0.66 [0.29, 1.49] NS
ALP (U/L)	M (IQR)	84 (68–123)	76 (59–96)	78 (60–101)	MW: *p* = 0.038
ALP (≥2 ULN)	N (%)	14/60 (23.3%)	28/152 (18.4%)	42/ 212 (19.8%)	OR = 1.35 [0.63, 2.76] NS

ULN, upper limit of the normal; CRP, C-reactive protein; ALT, alanine aminotransferase; AST, aspartate aminotransferase; GGT, gamma-glutamyl transferase; LDH, lactate dehydrogenase; CI, confidence interval; IQR, interquartile range; M, median (min:max); MW, Mann–Whitney test; OR, odds ratio; OR (95% CI) and *p*-value from Fisher test; NS, not significant.

**Table 5:** Univariate analysis of laboratory variables at discharge, non-survivors vs. survivors.

Variable		Non-Survivors N = 116 (21%)	Survivors N = 435 (79%)	Total N = 551	Statistics
D-dimer (mg/L)	M (IQR)	3.1 (1.5–5.2)	0.67 (0.38–1.3)	0.82 (0.4–2.1)	MW: *p* < 0.0001
D-dimer > 1 mg/L	N %	63/75 (84%)	118/350 (33.7%)	181/425 (42.6%)	OR = 10.32 [5.31, 19.76] *p* < 0.0001
CRP (mg/L)	M (IQR)	93 (43–200)	6.3 (2.1–17)	10 (2.8–43)	MW: *p* < 0.0001
CRP > 75 mg/L	N %	66/108 (61%)	19/375 (5%)	88/483 (17.6%)	OR = 29.44 [16, 51.8] *p* < 0.0001
Lymphocyte N × 10^3^/µL	M (IQR)	0.68 (0.36–1.2)	1.3 (0.83–1.9)	1.2 (0.7–1.8)	MW: *p* < 0.0001
Lymphocyte < 1 × 10^3^/µL	N %	72/109 (66%)	126/373 (33.8%)	198/482 (41%)	OR= 3.8 [2.41, 5.95] *p* < 0.0001
Ferritin (ng/mL)	M (IQR)	1218 (619–2163)	460 (269–746)	515 (294–746)	MW: *p* < 0.0001
Il-6	M (IQR)	128 (29–726)	5.5 (2–19)	7 (2.5–38)	MW: *p* < 0.0001
LDH (U/L)	M (IQR)	588 (413–846)	250 (206–311)	270 (213–270)	MW: *p* < 0.0001
LDH >400 U/L	N (%)	52/69 (75.4%)	30/265 (11.3%)	82/334 (24.5%)	OR = 23.96 [12.032, 44.83] *p* < 0.0001
AST (U/L)	M (IQR)	47 (29–121)	50 (27–86)	48 (28–89)	MW: NS
AST ≥ 2 ULN	N %	28/84 (33.3%)	60/336 (17.8%)	88/420 (21%)	OR = 2.3 [1.33, 3.93] *p* = 0.0022
AST ≥ 5 ULN	N %	13/84 (15.5%)	9/336 (2.7%)	22/420 (5.2%)	OR = 6.65 [2.66, 15.86] *p* < 0.0001
AST≥ 2 ULN and remdesivir use	N %	15/71 (21%)	26/216 (12%)	41/287 (14.3%)	OR= 1.97 [0.97, 3.85] NS
ALT (U/L)	M (IQR)	49 (29–112)	29 (20–51)	31 (21–59)	MW: *p* < 0.0001
ALT ≥ 2 UNV	N %	23/84 (27.4%)	28/336 (8.3%)	51/420 (12%)	OR = 4.14 [2.42, 7.48] *p* < 0.0001
ALT ≥ 5 UNV	N %	13/84 (15.5%)	4/336 (1.2%)	17/420 (4%)	OR = 15.2 [4.9, 43.4] *p* < 0.0001
ALT ≥ 2 ULN and remdesivir	N %	13/71 (18.3%)	17/216 (7.8%)	30/287 (10.5%)	OR= 2.62 [1.22, 5.75] *p* = 0.023
GGT (UI/L)	M (IQR)	177 (95–240)	133(87–237)	148 (87–236)	MW: NS
GGT ≥ 2 ULN	N (%)	13/17 (76.5%)	40/58 (68.9%)	53/75 (70.7%)	OR = 1.46 [0.43, 4.53] NS
GGT ≥ 5 ULN	N (%)	4/17 (23.5%)	40/58 (20.7%)	44/75 (58.7%)	OR = 1.03 [0.32, 3.48] NS
ALP UI/L	M (IQR)	127 (95–164)	84 (58–119)	103 (65–128)	MW: *p* = 0.0385
ALP ≥ 2 ULN	N (%)	1/9 (11%)	2/27 (7.4%)	3/36 (8.3%)	OR = 1.56 [0.09, 14.64] NS

ULN, upper limit of the normal; CRP, C-reactive protein; ALT, alanine aminotransferase; AST, aspartate aminotransferase; GGT, gamma-glutamyl transferase; LDH, lactate dehydrogenase; CI, confidence interval; IQR, interquartile range; M, median (min:max); MW, Mann–Whitney test; OR, odds ratio; OR (95% CI) and *p*-value from Fisher test; NS, not significant.

## Data Availability

Data are available on request due to restrictions, e.g., privacy or ethical. The data presented in this study are available on request from the corresponding author.

## Supplementary Materials

The following supporting information can be downloaded at: https://www.mdpi.com/article/10.3390/ph17010003/s1, Table S1: Spearman’s correlations (rho and *p* value) between liver parameters at admission (1), 7 days (2), and discharge (3).
