# Supplementary material for: Effects of COVID-19 on the Liver and Mortality in Patients with SARS-CoV-2 Pneumonia Caused by Delta and Non-Delta Variants: An Analysis in a Single Centre
Source: Pharmaceuticals (Basel). 2023 Dec 19;17(1):3. doi: 10.3390/ph17010003 (PMC10820137; doi:10.3390/ph17010003)
Supplement: Supplementary file 1 [file pharmaceuticals-17-00003-s001.zip › pharmaceuticals-2659235-supplementary.pdf]

Table S1. Spearman's Correlations (rho and p-value) between liver parameters at admission (1), 7 days (2) and discharge (3)

		AST1	ALT1	GGT1	AST2	ALT2	GGT2	AST3	ALT3	GGT3
**AST1**	Rho	**1.000**	**0.627****	0.155	**0.492****	**0.365****	0.178	**0.393****	**0.165****	0.251
	p-value	.	**0.000**	0.053	**0.000**	**0.000**	0.192	**0.000**	**0.006**	0.073
**ALT1**	Rho	**0.627****	**1.000**	**0.338****	**0.462****	**0.470****	**0.470****	**0.403****	**0.320****	**.360****
	p-value	**0.000**	.	**0.000**	**0.000**	**0.000**	**0.000**	**0.000**	**0.000**	**0.009**
**GGT1**	Rho	0.155	**0.338****	**1.000**	**0.236***	**0.271****	**0.860****	**0.322****	**0.237****	**0.817****
	p-value	0.053	**0.000**	.	**0.018**	**0.006**	**0.000**	**0.000**	**0.008**	**0.000**
**AST2**	Rho	**0.492****	**0.462****	0.236*	**1.000**	**0.638****	**0.410****	**0.598****	**0.259****	0.067
	p-value	**0.000**	**0.000**	0.018	.	**0.000**	**0.003**	**0.000**	**0.001**	0.722
**ALT2**	Rho	**0.365****	**0.470****	**0.271****	**0.638****	**1.000**	**0.306***	**0.321****	**0.573****	0.088
	p-value	**0.000**	**0.000**	**0.006**	**0.000**	**.**	**0.029**	**0.000**	**0.000**	0.638
**GGT2**	Rho	0.178	**0.470****	**0.860****	**0.410****	**0.306***	**1.000**	**0.361***	0.122	**0.840****
	p-value	0.192	**0.000**	**0.000**	**0.003**	**0.029**	.	**0.016**	0.430	**0.000**
**AST3**	Rho	**0.393****	**0.403****	**0.322****	**0.598****	**0.321****	**0.361***	**1.000**	**0.548****	**0.463****
	p-value	**0.000**	**0.000**	**0.000**	**0.000**	**0.000**	**0.016**	**.**	**0.000**	**0.001**
**ALT3**	Rho	**0.165****	**0.320****	**0.237****	**0.259****	**0.573****	0.122	**0.548****	**1.000**	**0.419****
	p-value	**0.006**	**0.000**	**0.008**	**0.001**	**0.000**	0.430	**0.000**	**.**	**0.002**
**GGT3**	Rho	0.251	**0.360****	**0.817****	0.067	0.088	**0.840****	**0.463****	**0.419****	**1.000**
	p-value	0.073	**0.009**	**0.000**	0.722	0.638	**0.000**	**0.001**	**0.002**	**.**

**. Correlation is significant at the 0.01 level (2-tailed) and * at the 0.05 level (2-tailed).

ALT, alanine aminotransferase; AST, aspartate aminotransferase; GGT, gamma glutamyl transferase.
